# Barriers and facilitators to provide effective pre-hospital trauma care for road traffic injury victims in Iran: a grounded theory approach

**DOI:** 10.1186/1471-227X-10-20

**Published:** 2010-11-08

**Authors:** Hassan Haghparast-Bidgoli, Marie Hasselberg, Hamidreza Khankeh, Davoud Khorasani-Zavareh, Eva Johansson

**Affiliations:** 1 Division of Global Health, Department of Public Health Sciences, Karolinska Institute, Stockholm, Sweden; 2 The Swedish Research School for Global Health, Partnership between Umeå University and Karolinska Institute, Sweden; 3 Health Management and Economics Research Centre, Faculty of Management and Informatics, Isfahan University of Medical Sciences, Isfahan, Iran; 4 Department of Nursing, University of Social Welfare and Rehabilitation, Tehran, Iran; 5 Department of Public Health, Urmia University of Medical Sciences, Urmia, Iran; 6 Nordic School of Public Health, Gothenburg, Sweden

## Abstract

**Background:**

Road traffic injuries are a major global public health problem. Improvements in pre-hospital trauma care can help minimize mortality and morbidity from road traffic injuries (RTIs) worldwide, particularly in low- and middle-income countries (LMICs) with a high rate of RTIs such as Iran. The current study aimed to explore pre-hospital trauma care process for RTI victims in Iran and to identify potential areas for improvements based on the experience and perception of pre-hospital trauma care professionals.

**Methods:**

A qualitative study design using a grounded theory approach was selected. The data, collected via in-depth interviews with 15 pre-hospital trauma care professionals, were analyzed using the constant comparative method.

**Results:**

Seven categories emerged to describe the factors that hinder or facilitate an effective pre-hospital trauma care process: (1) administration and organization, (2) staff qualifications and competences, (3) availability and distribution of resources, (4) communication and transportation, (5) involved organizations, (6) laypeople and (7) infrastructure. The core category that emerged from the other categories was defined as "interaction and common understanding". Moreover, a conceptual model was developed based on the categories.

**Conclusions:**

Improving the interaction within the current pre-hospital trauma care system and building a common understanding of the role of the Emergency Medical Services (EMS) emerged as key issues in the development of an effective pre-hospital trauma care process.

## Background

Road traffic injuries (RTIs) are a major public health problem globally causing more than a million deaths and almost 50 million injuries every year [1]. Low and middle income countries (LMICs) account for 90% of Disability Adjusted Life Years (DALYs) lost and for 90% of the deaths from road traffic crashes [1-3]. As the majority of trauma deaths in LMICs occur in the pre-hospital setting [4-6], it is suggested that improvements in pre-hospital trauma care can contribute to a decrease in crash-related mortality and morbidity [4,7-11].

The pre-hospital trauma care process consists of six key steps: detection, reporting, response, on-scene care, care in transit and transfer to definitive care [12] (The six steps inspired from the Emergency Medical Services-EMS-symbol or so-called 'Star of Life' symbol created by the US National Highway Traffic Safety Administration which presents six EMS functions[13]). The essential elements of a pre-hospital trauma care system include prompt communication and activation of the system, timely response of the system, correct assessment and efficient treatment, and prompt transport of injured people to a formal health-care facility when necessary [14]. EMS is responsible for providing pre-hospital trauma care in many countries and can be described as the link between pre-hospital care and care at the hospital. The World Bank [15] has presented an overview of the role of EMS and key issues when providing trauma care for injured people (see Table 1).

**Table 1:** Overview of Emergency Medical Services

	*Acute event*	*On-site management*	*Transportation*	*Health facility care*
**Role of EMS**	Recognition	Triage, stabilization, or both	Safe and efficient transportation	Prompt, appropriate, and quality care
**Key issues**	Surveillance, identification	Trained personnel, equipment	Safe transportation, equipment, referral system	Personnel, equipment, organization of services

Source: Jamison D.T, Breman, Joel G, et al, 2006

Many LMICs have insufficient pre-hospital trauma care [1,16,17], few victims receive treatment at the crash scene and even fewer receive safe transport to the hospital by an ambulance. Injured people are usually cared for and transported to the hospital by relatives, untrained laypeople or drivers of commercial vehicles [1,10,16-18].

Iran with one of the highest RTI death rates (annually with over 27,000 deaths and about 0.8 million injured) in the world [19-21] has a situation similar to that described above. Studies in Iran have shown that about 60% of the deaths occurred at the crash scene or on the way to hospital and more than 30% at the hospital [6,20,22]. Furthermore, a survey in 2002 indicates that only 14% of the injured people are transported to hospitals by ambulance and only 10% are rescued by trained personnel [20]. In order to reduce crash consequences, EMS capabilities in terms of human and physical resources have improved substantially during recent years [23,24], but the statistics for crash-related mortality and morbidity do not show a noticeable decrease [24].

Few studies have been done on trauma care for injured people in Iran and those that have been conducted have mainly focused on evaluating pre-hospital time intervals and quality of trauma care provided in the hospitals [22,25-27]. One exception is a recently published study about the barriers to post-crash management in Iran [24], where the authors mainly discussed the role of laypeople and the involvement of other organizations at the crash scene. Studies conducted on trauma care in other LMICs have mainly concentrated on availability of resources and effective interventions done in pre-hospital settings, especially training of laypeople and EMS personnel [4,8,18,28-32]. With the aim of exploring the process of pre-hospital trauma care for RTI victims in Iran and identifying potential areas for improvements, the current study explores different aspects of providing pre-hospital trauma care based on the experience and perceptions of pre-hospital trauma care professionals.

## Methods

A grounded theory approach was used for the collection and analyses of data. According to Strauss and Corbin [33], findings grounded in data are likely to offer insight, enhance understanding, and provide a meaningful guide to action. This method is suitable when relatively new areas are to be discovered or if one desires to explore a known area from a fresh perspective [33,34].

### Study setting

This study was conducted among pre-hospital trauma care professionals, mainly from Tehran, the capital city and the largest city in Iran with a population of around 13 million [35]. The total number of RTI deaths in Tehran in 2006 was 2645 (20 per 100,000) [36].

**Figure 1:**
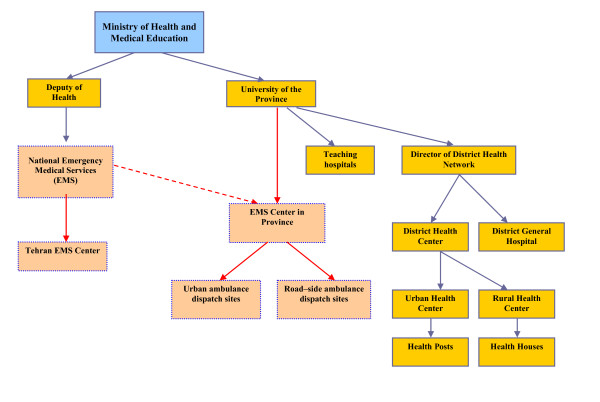
**Position of the EMS in the Iranian Health care system structure**.

The EMS in Iran, which is mainly based on a Basic Life Support (BLS) system [9], is centralized under the Ministry of Health. Provincial centres are affiliated to the Medical Sciences and Health Services University in each province (Figure 1). In Tehran city, pre-hospital trauma care is provided by the local EMS center that is directly governed by the national EMS center in Ministry of Health. In 2006 the Tehran EMS centre had 138 ambulance dispatch sites (urban and road-side), 275 ambulances (which were mainly equipped with BLS instruments) and 1614 staff (including physicians, nurses, emergency medical technicians and other staff) [36].

The EMS center in Tehran receives more than 1000 calls each day [25]. The operators, who answer the calls in the EMS central dispatch, are usually trained nurses. For each incoming call the operator determines whether the situation needs the dispatch of an ambulance or not. Once the decision to dispatch an ambulance is made, the operator informs the ambulance dispatch site closest to the crash scene to help the victim [25]. Moreover, there are a number of general physicians in the central dispatch who provide medical consultation for people who call EMS and also give medical advice to the technicians who treat victims at the crash scene or on the way to the hospital. According to the referral system, Tehran (like other big cities) is divided into several regions by the EMS. Each region has one trauma hospital and if a crash occurs in that region, the ambulance should transfer the patient to that specific hospital.

### Study participants and data collection

The participants of the study consisted of fourteen male and one female health professionals, all have at least three years experience in pre-hospital trauma care (including four physicians, eight nurses and three emergency medical technicians working as ambulance staff, manager or adviser in Tehran EMS center (ten participants) and national EMS center (three participants) and Oroumiyeh EMS center (two participants). The reason for the high number of male participants was that ambulance staff and administrative staff are usually men in Iran. Purposeful sampling was used for the initial interviews and according to the emerging codes and categories data were collected by means of theoretical sampling. Participant selection, data collection and data analysis continued until theoretical saturation was reached and a rich description of experience had been obtained.

In-depth interviews in Persian were used for data collection. Each interview began with general questions about the participants' own experiences of the pre-hospital trauma care process for RTI victims and their perceptions about "factors affecting an effective pre-hospital trauma care process". Probing questions were also used to clarify information and gain additional data. The interviews lasted from 20 to 100 minutes (no association between interview time and profession of interviewee was observed). Seven interviews were done between January and April 2009 by the first author (H.H.B.) and eight interviews were conducted between March and December 2007 by one of the other researchers within the research team (D.K.Z).

The research team is a multi-professional one including both male and female researchers with different backgrounds from Iran and Sweden; one sociologist (M.H) with experience of injury research, two nurses (E.J and H.K) with expertise in public health and qualitative research, one MD (D.K.Z) working with road traffic injury studies and one health economist (first author, H.H.B) active in studies of road traffic injury and trauma care.

### Data analysis

All interviews were recorded, transcribed verbatim and analyzed using constant comparative method [33]. Data collection and data analysis took place simultaneously and an initial analysis of each interview was made before the next interview and if some important issues emerged they were then brought up in the next interview. Accordingly, each interview provided the direction for the next one.

Open, axial and selective coding was applied to the data [33]. Open coding involved a line by line analysis and labeling and grouping of the data into categories and sub-categories. At the open coding stage about 500 substantive codes and 12 categories were explored. Axial coding involved further conceptualization of the categories by specifying the relationships between them and by integrating them into a new form. Finally the number of categories was reduced and major new categories were generated. Selective coding resulted in one core category which related to all other categories. All the analyses were done by the first author (H.H.B.) in collaboration with the research team.

### Rigour

Regarding trustworthiness, credibility was ensured through constant comparison, triangulation, member check, and peer review. Constant comparison was done by returning to the data several times during the analysis to verify and develop categories. Seven of the participants were contacted after the analysis and were given a summary of the primary results to determine whether these results were in accordance with their experiences (member check). As a further validity check, some parts of all the transcripts and the preliminary sets of codes and categories were checked by two experts in qualitative method within the research team and also by the other co-authors (peer review). Moreover, triangulation of researchers in the research team helped to take into account different perspectives when analyzing the data.

### Ethical considerations

Verbal consent was obtained and all participants were informed that they could refuse to participate or withdraw from the interviews at any time. Ethical clearance of the study was obtained from the National Ethics Committee of Ministry of Health in Iran.

## Results

In the process of data analysis, seven categories finally emerged: (1) administration and organization, (2) staff qualifications and competences, (3) availability and distribution of resources, (4) communication and transportation, (5) involved organizations, (6) laypeople and (7) infrastructures. We divided these categories into factors inside the EMS and factors outside the EMS. The core category that was related to all the other categories was defined as "interaction and common understanding".

**Figure 2:**
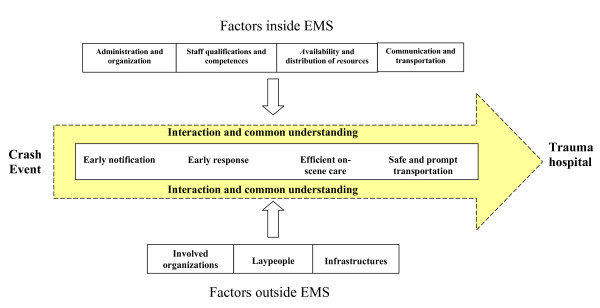
**Model of factors affecting the Optimal Pre-hospital Trauma Care Process**.

We generated a model grounded in our data which illustrates factors that can influence the pre-hospital trauma care process (Figure 2). In the model, the pre-hospital trauma care process is illustrated as an arrow and is divided into four main stages (inspired from available knowledge in the literature [12-15]): Early notification, early response (or dispatch), efficient on-scene care and safe and prompt transportation.

Each category by itself or in interaction with others can facilitate or inhibit an optimal pre-hospital trauma care process by influencing each of the main stages of the process. In the following we present the categories, which emerged from the data, considering their effects on the pre-hospital trauma care process.

### Factors inside the EMS

#### Administration and organization

The participants emphasized that certain aspects of the current administration and organization contribute to an inefficient pre-hospital trauma care process. Factors such as existing misconceptions about the EMS, inappropriate management, inefficient structure and rules and regulation were brought up by the participants.

Misconceptions regarding the content and role of the EMS among health policy makers and EMS managers were mentioned as important factors affecting the development of the EMS in the country.

"Because EMS is free of charge and does not generate income for the health system and it is merely a consumer of health care resources, the health care managers often look upon EMS as is an expensive part of the system and do not focus on the development of EMS". (Participant 1)

"Some EMS managers say that if we have more transportation to hospital, we would have a more dynamic EMS.... When the view of managers is narrowed down to the transfer of victims then improving the quality of services and other important issues in EMS won't be emphasized". (Participant 1)

Regarding the structure of the EMS, the participants pointed out that due to the independent role of the Medical Universities in the provinces, the structure of the EMS varies between different provinces. This leads to inappropriate coordination between EMS centers, especially in mass trauma situations, and also, reduces the authority of the national EMS center in relation to the provincial EMS centers.

"We in the central EMS prepare national policies and plans for the whole country, but all medical universities (in the provinces) are independent and have a different structure. The EMS centers in some universities are governed by the Chancellor and in others by the Deputy of Treatment..., sometimes they decide to not implement our policies or take a long time to implement our guidelines". (Participant 4)

The participants also expressed the opinion that some of the existing official rules and regulations hinder an effective pre-hospital trauma care. Restrictions on the employment of experienced physicians and the limited responsibility of experienced nurses to treat patients were important examples mentioned by the participants.

"The rules say that the EMS can't employ physicians, and then leaving us with two options: to use other medical professionals instead or to use newly graduated physicians without any experience in trauma care". (Participant 3)

Other issues related to management were limited financial resources, suboptimal and unstable management, low economic incentives for staff, lack of protocols and inadequate training plans for staff.

"...the EMS managers are usually not familiar with the EMS standards and principles. They don't know the mission and philosophy of the EMS. They can't manage EMS without this knowledge". (Participant 1)

"A constant turnover of managers is another issue. We have had nine managers over the past nine years. Each one was replaced by another person after having gained experience about the EMS and making some new policies or plans". (Participant 3)

Low economic incentives for the staff resulted in a lack of motivation among medical professionals to work in the EMS, and leading to EMS managers employing non-medical staff instead. Low economic compensation also led to a high work load among existing EMS staff.

"Because of financial problems, a lot of our staff are working on two shifts. Some of them are working at the hospital too and have two jobs. They are tired when they get home, which creates problems in their families. Their fatigue can also affect their performance on their next shift". (Participant 1)

"Working in the EMS is stressful and hard, but the salary is very low for a professional and because of that professionals are not willing to work in the EMS". (Participant 5)

#### Staff qualifications and competences

Most participants were concerned with the shortages of professional medical staff and the inadequate skills and knowledge of the current staff in EMS. Inappropriate training plans about pre-hospital trauma care and out of date, unpractical and inadequate training courses were mentioned as the main reasons for inadequate skills and knowledge among the staff, although they noted that EMS educational plans have improved considerably during recent years, especially with the assistance of Emergency Medicine specialists. Malpractice, conflicts among staff members and interference from untrained laypeople were perceived to be the consequences of the inadequate skills and knowledge of staff.

"We employ nurses and physicians without any basic training or practical experience of trauma care. As a consequence of the shortage of professional medical staff and the fact, that they are not willing to work in the EMS, we have in recent years employed a lot of non-medical staff and trained them in basic first aid". (Participant 1)

"We have a lot of useful training courses but management doesn't ensure that these courses are practical. New text books are used for the training courses, but in reality we use the same procedures as we always have". (Participant 2)

Conflict between the ambulance staff and consultant physicians was another issue that was discussed by many participants. They explained that there is a wall of mistrust between ambulance staff and consultant physicians.

"Consultant physicians don't trust the ambulance staff reports and physical examinations. Ambulance staff with different levels of experience and education call them and each of them gives a different report and information about the patient. If you were one of physician, you wouldn't trust the reports, and you wouldn't take the risk. This is the case for the consultant physicians too. They don't ask for detailed reports, their orders are wrong or they order the same things for all cases". (Participant 1)

Moreover, the participants reported that the ambulance staffs often misjudge emergency cases due to the high number of non-emergency dispatches. The following comment reflects this:

"Since the staff have a lot of non-emergency dispatches during the day, they think that every case is non-emergency like most of the cases and if a real emergency happens they are not mentally and practically prepared". (Participant 1)

According to the participants, non-emergency dispatches resulted from non-emergency calls from the public, calls from the police about crashes without injury and also inappropriate screening of calls in the central dispatch. Fatigue and dissatisfaction of the staff and also a risk of missing the actual emergency cases that need trauma care are described as the main consequences of a high rate of non-emergency dispatches.

#### Availability and distribution of resources

Deficiency and maldistribution of resources were viewed as an important barrier for providing effective pre-hospital trauma care. The factors that contributed to resource deficiency and inadequacy were described as short supply of professional staff, ambulances and dispatch sites, lack of necessary equipment in the ambulances (e.g. defibrillator and monitoring equipments), lack of rescue equipment in the ambulances, and lack of some drugs (e.g. painkillers). Some other concerns were the long distances between ambulance dispatch sites (which affects response time), and substandard road-side dispatch sites.

"At some crashes, when we reach the scene we see that the victim is trapped in the car and we have to call the Fire Department because we don't have rescue equipment to help the victim. This is a time loss which is critical for the victim's life". (Participant 10)

Inappropriate distribution of the resources was another important barrier to providing effective pre-hospital trauma care raised by the participants.

"One of the most important problems that we have is a mismatch between the available number of ambulance dispatch sites, ambulances, staff and the population size and population density....these facilities are not sufficient in relation to the number of accidents that occur and the number of black spots". (Participant 1)

#### Communication and transportation

According to the participants, an inappropriate communication system, and an ineffective medical direction and referral system are major barriers when providing care on the scene or when transporting the victims to the hospital. Large areas with no radio coverage, an insufficient number of radio channels and a shortage of equipment (e.g. lack of repeater and backup system) were some of the limitations of the radio communication system.

"The number of radio lines is limited and the coverage is not good. We have some areas in the city, where we have no coverage and then we have to call to the nearest dispatch center to send our message to the central dispatch center". (Participant 2)

"Sometimes the line is very busy and there is a lot of noise so we can't contact the central dispatch center or report to the consultant physicians". (Participant 1)

Inefficient medical direction was another important issue, which was considered as being a consequence of communication system limitations, poorly experienced consultant physicians and staff with inadequate skills. Moreover, even if taking medical advice from the consultant physicians is obligatory for all cases, the participants implied that for many cases it is not needed and this is more a waste of time which can be critical for the patient's health outcome.

"We have telephone consulting with physicians, but in practice it's not efficient because of the technical problems with the communication system. We have a lot of ambulance dispatches every day and the radio lines and consultant physicians are very busy and there is not a special line for the consultant physicians and we can't wait long for that... sometimes the services that we provide for the victims are better than the consultant physicians' advice". (Participant 2)

Moreover, the participants explained that the referral system has some limitations that may contribute to a delayed transportation of the victim to the hospital. One of the major limitations identified was using out of date maps in the referral system something that become even more important in the absence of Satellite Navigation (GPS). Furthermore, the difficulties in contacting hospitals due to the limitations of the communication system have a major effect on the referral system.

"The referral system has a lot of problems. The streets have changed, and since we don't have GPS, we can't track our ambulances. The maps we are using are old and not updated. This system is ineffective, not only for traumas but also for other specialties". (Participant 1)

### Factors outside the EMS

#### Involved organizations

In addition to the EMS, there are three other organizations involved in the management of a crash and the rescue of crash victims in Iran: the Police, the Red Crescent and the Fire Department. The participants believed that poor coordination and cooperation between these organizations and the EMS and the insufficient knowledge and skills regarding the rescue of victims and managing the crash are important obstacles to providing prompt and effective pre-hospital trauma care at the crash scene and when transporting the victims to hospital. They explained that each organization arrives at the crash scene at different times and there is no communication or a common telephone line between these organizations that can be used for coordination and information exchange. The participants pointed out that the Red Crescent and the Fire Department have rescue teams who are responsible for rescuing victims in some specific situations such as when a victim is trapped in a car. However, these teams sometimes arrive late on the scene and often try to rescue the victims in an unsafe way. The teams were not considered qualified to provide medical care for victims, this is especially true for the Red Crescent staff, who mostly are volunteers. Sometimes, the delayed arrival of the police, combined with a lack of cooperation in ensuring a safe and secure environment for EMS staff constitutes another important barrier to timely and effective trauma care.

"The police staff are usually bystanders at the crash scene like other laypeople. They only do paper work related to the crash (take statements)". (Participant 2)

"The Red Crescent staff are mainly volunteers and they are not usually qualified to treat some types of trauma patients, they take action because they usually are the first on the scene.". (Participant 1 and participant 5)

#### Laypeople

The involvement of laypeople at the crash scene was perceived as negative due to reasons such as providing incomplete or wrong information, and emotional reactions and conflicts with the EMS personnel. The participants expressed that interference from laypeople and their forming a crowd at the crash scene may result in wasting critical time in providing effective care and also, in some circumstances, may contribute to secondary injuries for the victims and even lead to a new crash. They pointed out that factors such as cultural values and beliefs (including: humanitarian assistance, willingness to help, curiosity and excitement), lack of knowledge together with the late arrival and lack of competence of EMS staff and laypeople's mistrust in them are factors leading to laypeople's interaction or interference at the crash scene.

"Laypeople interfere with the EMS technicians at the scene and distress them so they can't focus carefully on their work and they have to take victims without doing their routine examinations ..." (Participant 1)

"I think one of the reasons that laypeople interfere in the crash scene is that they don't know what emergency care means and what EMS is doing". (Participant 9)

"Wrong addresses by the public are one of our problems that waste our time when finding the correct location". (Participant 2)

Furthermore, lack of public educational plans about providing first aid at the crash scene, unclear roles of the involved organizations and also laypeople at the crash scene were emphasized as important issues. The participants indicated that there is inadequate collaboration and interaction between EMS and the media concerning public education. They also noted that the role of other involved organizations about public education (including laypeople) is not clear either.

"One of the problems that we have is lack of public education about EMS in the country. A lot of people are not familiar with EMS and its role in emergency situations" (Participant 11)

"There is no interaction between the EMS and the media about public education. This is because EMS managers don't believe that public education is a part of the EMS mission". (Participant 1)

#### Infrastructure

A number of factors were mentioned as potential obstacles to an efficient infrastructure for pre-hospital trauma care such as lack of GPS system, sub-standard road infrastructures (including lack of an emergency lane in cities and free-ways outside cities), lack of infrastructures for helicopter ambulances in the big cities, and an inadequate telecommunication system.

"On the free-ways outside the cities there is no special lane for emergency services and no time standard for access to an under-pass or slip road [to change direction on the free-way]. On some freeways we have to drive 3 to 4 minutes to reach a slip road and in other places 6 to 7 minutes". (Participant 5)

"One problem that we have is sub-standard roads. We don't have special lanes for emergency services in all streets in the city and because of that it very often takes a long time to reach the crash scene due to the traffic". (Participant 9)

#### Core category: Interaction and common understanding

Interaction and common understanding was identified as the core category in this study. This category was visible among all actors involved inside and outside the EMS system, including health policy makers, managers and staff within the EMS, laypeople, actors within involved organizations on the crash scene, and actors within other influential sectors outside EMS.

The participants indicated that the misconceptions about the role of EMS among health policy makers and EMS managers may result from their inadequate knowledge about EMS principles and standards. Moreover, they stated that this was aggravated by the weakness of the current EMS structure which hinders better coordination and interaction between different actors and EMS centers across the country. According to the participants, the development of EMS (including increasing its resources and quality improvement in EMS) requires that the health policy makers and EMS managers deepen their understanding of the EMS.

Participants believed that the conflicts and inadequate interaction between staff in general was caused by a poor communication system and their inadequate knowledge and skills. They also argued that lack of documented protocols about individuals responsibilities led to misunderstandings among staff about their respective duties.

Furthermore, the participants reported that the role of different organizations involved in the management of a crash is not clear and also that there is inappropriate communication, information and knowledge exchange between these organizations. These facts hinder an effective interaction between them, which makes it difficult to reach a common understanding about their respective roles in the crash scene. This is also true for other sectors affecting the EMS, especially the Road Administration Offices. The participants believed that inadequate interactions between these sectors and the involved organizations results in the failure to identify certain needs that are essential for the infrastructure like emergency lanes.

The negative involvement of laypeople perceived by the participants was mainly explained by cultural values and beliefs and laypeople's lack of knowledge about their role and how they should interact at the crash scene.

Suggestions that came up for improving interaction and building a common understanding among different actors, mainly focused on ways to improve communication and information exchange, improve coordination, and increase the knowledge and skills of the actors. The participants suggested a reform of the current EMS structure and its rules and regulations in order to facilitate better communication and coordination between different EMS centers across the country. To improve the knowledge and skills of staff, along with communication and interaction through multidisciplinary meetings, were other suggestions by the participants.

Participants universally acknowledged the important role of the police for coordinating post-crash activities through notifying crash occurrences to EMS and other rescue organizations. But they emphasized the importance of establishing a committee or authority responsible for coordinating all post-crash management activities. This authority should provide a clear definition and allocation of roles and responsibilities for all involved organizations so they not only fully understand their own roles when a crash occurs but also their relation to those of other organizations and actors. An integrated communication system with a single three digit emergency number for the public and including all involved organizations at national level and common dispatch sites at local levels were other suggestions for improving coordination and interaction between the involved organizations.

Moreover, almost all participants emphasized public education campaigns using the mass media, especially TV, and also educational plans for special target groups as the most efficient way of improving public knowledge.

## Discussion

Based on the findings of the current study, two groups of factors can inhibit or facilitate an efficient pre-hospital trauma care process in an Iranian context: factors inside the EMS and factors outside the EMS. Administration and organization, staff qualifications and competences, availability and distribution of resources and communication and transportation are factors inside the EMS and involvement of other organizations, laypeople and the general infrastructure are factors outside the EMS that influence the process. Interaction and common understanding was identified as the core category which emerged from the gathered information in different ways such as misconceptions, inadequate knowledge, poor cooperation and coordination, poor communication and information exchange, different cultural values and beliefs, interference and conflicts.

The findings from the current study, are to some extent, consistent with a study by Mock et al [4], which indicates that human resources, physical resources and organization and administration are critical weak points in trauma systems, especially for hospital trauma care in LMICs. Based on the findings from the current study and another study done in 2000 in Iran [25] shortages of professional staff, ambulances and dispatch sites were important barriers to providing effective pre-hospital trauma care. Moreover, the inappropriate distribution of the resources is another issue that was brought up in the current study that could be explained by the general shortage of resources. But in general, the issue of shortage of resources was not seen as a major problem compared to other issues.

Based on the findings of the current study, inadequate knowledge and skills of staff was another important barrier. This is in line with a study from Ghana and Mexico [4]. The main reasons for this problem in the context of the current study could be explained by the inappropriate practical education, poor educational plans and insufficient motivation among staff to attend training courses. This differs from other LMICs which mainly rely on staff and volunteers with only on-the-job training and without any formal training, such as the Emergency Medical Technician (EMT) certification [4,37]. The high numbers of formally educated staff in Iran make it possible to develop a comprehensive educational plan for pre-hospital trauma care and to link staff's education and reality (practice) by implementing evidence-based training courses. This can be done by the help of Emergency Medicine Specialists who are perceived to have had an important role in improving the quality of trauma care in recent years [38]. Training staff in BLS, such as Pre-hospital Trauma Life Support (PHTLS) program [8,31,32] and providing EMT certification [37] have proven to be effective in LMICs. For example, in Mexico [8] an increased number of ambulance dispatch sites and the establishment of regular PHTLS courses for ambulance attendants decreased the mortality among transported trauma patients from 8.2% to 4.7%. Similar improvements in trauma care and a decrease in mortality (from 15.7% to 10.6%) was seen in Trinidad when they established regular PHTLS [31,32]. On the other hand, the effect of training of staff in Advanced Life Support skills to improve patient's survival is not clear [1,18,39].

Inappropriate administration and organization was identified as one of the critical barriers to an effective pre-hospital trauma care in the current study because it influences all the other essential components of the EMS. In contrast to Mock et al[4], who mainly focused on supply and utilization procedures, the findings from the current study are mainly concerned with issues such as the inappropriate structure of the EMS, misconceptions among health policy makers and EMS managers and a low level of motivation among staff. The problem of misconceptions about the EMS among health policy makers has also been discussed in other studies from LMICs [15]. This is also a problem between staff categories on lower levels. They do not seem to be fully aware of each other's roles.

The current study showed that inappropriate communication network, ineffective medical consultation and inefficient referral system are other important factors that hinder the provision of effective trauma care on the scene or when transporting the victims to hospital. These factors, which are related to each other, could be facilitated by a combination of short and long-term interventions. Some examples of these facilitating factors could be the use of new technologies for improving communication network in addition to increasing the number of radio channels, to employ experienced physicians for medical consultation and to provide appropriate trauma care courses for them and also updating maps related to the referral system and establishing GPS.

The present study is consistent with another study done in Iran focusing on disaster management [40] indicating that poor coordination and cooperation between involved organizations is a major barrier to an effective trauma care system. Integrated emergency dispatch or central call reception is one of the strategies recommended by World Health Organization (WHO) [17] for enhancing coordination between these organizations. The WHO [17] has also suggested that since the police and firefighters often arrive at the crash scene before EMS personnel, they need to be trained in BLS skills.

The involvement of laypeople was perceived as negative in the current study. This corroborates with a recent study done by Khorasani et al in Iran [24]. Cultural beliefs, lack of knowledge about emergency care and first aid and lack of public education programs seem to be the main reasons for this negative involvement. Laypeople are first responders in many countries and they are often trained to provide basic first aid for the victims before the arrival of more formally trained rescuers [17]. According to WHO [17], the media (especially TV in the Iranian context) can be used to train the public to recognize emergency medical situations, to call for help, and how to provide first aid. Training specific target groups, such as public car drivers, official staff, soldiers, high school students and volunteers, is another efficient way to improve pre-hospital trauma care. Several studies in LMICs [28-30], especially in settings with a high burden of injuries, have demonstrated the effectiveness of training laypeople in first aid. For example, training laypeople and paramedic staff in providing first aid in mine-infested areas of Iraq and Cambodia showed that mortality among severely-injured persons decreased from 40% to 9% [30]. However, a similar study in a rural area of Iran showed no difference in mortality among patients as a results of first responders (laypeople and paramedic) training although the physiological status of the transported victims improved [41].

More research needs to be done about the effectiveness of educational programs, especially regarding what type of intervention can be used for different types of injuries and in various settings. The educational programs proposed by WHO [17] included: basic first aid, Cardio Pulmonary Resuscitation, triage, basic principles of safe rescue and safe transportation to the hospital. However, in countries, such as Iran, with a high number of head injuries due to motorcycle crashes [20,22,23,26] it has to be discussed what type of education could be effective.

### Implications

The model developed in the current study can hopefully contribute to a better understanding of factors influencing the pre-hospital trauma care process and also provide useful information for policy making and development of the EMS system. The results can also generate new hypotheses within this research area.

### Strengths and limitations

The qualitative approach was used to explore the experience of pre-hospital trauma care professionals about providing pre-hospital trauma care for RTI victims in a middle income setting. This study is one of the few studies on trauma care that has employed this approach.

Different methods, including constant comparison method, member check, and peer review were used to increase the credibility and the consistency of the findings and the model that was developed. The model is regarded as a substantive model representing the context of the study setting. Being substantive, the model however, may be applied to similar settings, but more research is needed to identify applicable evidence.

One potential limitation of the current study is that the data collection was done by two different interviewers and at two points of time. This could also be seen as strength of the study since both interviewers were involved in the whole process of conducting the study.

Furthermore, the current study focused on experiences and perceptions of staff and professionals working in the EMS and do not reflect the views of other personnel involved in post-crash management. Further research needs to be done on other actors that are involved in the process of providing pre-hospital trauma care such as actors outside the EMS system and health policy makers within the Ministry of Health and the Medical Universities.

## Conclusions

The study illustrates the major barriers to and facilitators for providing effective pre-hospital trauma care for RTI victims in a middle income setting with rapidly increasing motorization. Based on the study findings, improving the interaction within the current pre-hospital trauma care system and building a common understanding of the role of the EMS emerged as key issues in the development of an effective pre-hospital trauma care system.

## List of Abbreviations

RTIs: Road traffic injuries; LMICs: Low- and middle-income countries; EMS: Emergency medical services; DALYs: Disability adjusted life years; BLS: Basic life support; GPS: Satellite navigation system; EMT: Emergency medical technician; PHTLS: Pre-hospital Trauma Life Support; WHO: World Health Organization

## Competing interests

The authors declare that they have no competing interests.

## Authors' contributions

HHB was involved in the study conception and design, data collection, analysis, revision, editing and manuscript writing. MH participated to the study conception and design, writing-up and finalization of the manuscript. HK was involved in the conception and design of study and took an active part in the data analysis and results interpretation. DKZ contributed to the data collection and helped to analyze and interpret the data and to write the manuscript. EJ participated to the study design, analysis and results interpretation and writing-up of the manuscript. All authors read and approved the final manuscript.

## Pre-publication history

The pre-publication history for this paper can be accessed here:

http://www.biomedcentral.com/1471-227X/10/20/prepub
